# Cerebral autoregulation derived blood pressure targets in elective neurosurgery

**DOI:** 10.1007/s10877-023-01115-0

**Published:** 2024-01-19

**Authors:** Erta Beqiri, Marta García-Orellana, Anna Politi, Frederick A. Zeiler, Michal M. Placek, Neus Fàbregas, Jeanette Tas, Veerle De Sloovere, Marek Czosnyka, Marcel Aries, Ricard Valero, Nicolás de Riva, Peter Smielewski

**Affiliations:** 1 Brain Physics Laboratory, Division of Neurosurgery, Department of Clinical Neurosciences, University of Cambridge, Cambridge, UK; 2 Neuroanesthesia Division, Anesthesiology Department, Hospital Clinic de Barcelona, Universitat de Barcelona, Barcelona, Spain; 3 Department of Anaesthesiology, Intensive Care and Pain Medicine, Kepler Universitätsklinikum, Neuromed Campus, Linz, Austria; 4 Department of Anesthesiology, Intensive Care and Pain Medicine, Milano Bicocca University, San Gerardo Hospital, Monza, Italy; 5 Section of Neurosurgery, Department of Surgery, Rady Faculty of Health Sciences, University of Manitoba, Winnipeg, Canada; 6 Department of Human Anatomy and Cell Science, Rady Faculty of Health Sciences, Univesity of Manitoba, Winnipeg, Canada; 7 Biomedical Engineering, Price Faculty of Engineering, University of Manitoba, Winnipeg, Canada; 8 Division of Anaesthesia, Department of Medicine, University of Cambridge, Cambridge, UK; 9 School for Mental Health and Neuroscience (MHeNS), University Maastricht, Maastricht, The Netherlands; 10 Department of Intensive Care, Maastricht UMC, Maastricht, The Netherlands; 11 Department of Anesthesiology, University Hospitals Leuven, Louvain, Belgium

**Keywords:** Individualised blood pressure, Intraoperative, Lower limit of autoregulation, Neurosurgery, Optimal blood pressure

## Abstract

Poor postoperative outcomes may be associated with cerebral ischaemia or hyperaemia, caused by episodes of arterial blood pressure (ABP) being outside the range of cerebral autoregulation (CA). Monitoring CA using COx (correlation between slow changes in mean ABP and regional cerebral O_2_ saturation—rSO_2_) could allow to individualise the management of ABP to preserve CA. We aimed to explore a continuous automated assessment of ABP_OPT_ (ABP where CA is best preserved) and ABP at the lower limit of autoregulation (LLA) in elective neurosurgery patients. Retrospective analysis of prospectively collected data of 85 patients [median age 60 (IQR 51–68)] undergoing elective neurosurgery. ABP_BASELINE_ was the mean of 3 pre-operative non-invasive measurements. ABP and rSO_2_ waveforms were processed to estimate COx-derived ABP_OPT_ and LLA trend-lines. We assessed: availability (number of patients where ABP_OPT_/LLA were available); time required to achieve first values; differences between ABP_OPT_/LLA and ABP. ABP_OPT_ and LLA availability was 86 and 89%. Median (IQR) time to achieve the first value was 97 (80–155) and 93 (78–122) min for ABP_OPT_ and LLA respectively. Median ABP_OPT_ [75 (69–84)] was lower than ABP_BASELINE_ [90 (84–95)] (*p* < 0.001, Mann-U test). Patients spent 72 (56–86) % of recorded time with ABP above or below ABP_OPT_ ± 5 mmHg. ABP_OPT_ and ABP time trends and variability were not related to each other within patients. 37.6% of patients had at least 1 hypotensive insult (ABP < LLA) during the monitoring time. It seems possible to assess individualised automated ABP targets during elective neurosurgery.

## Introduction

The approach of individualizing blood pressure targets to preserve cerebral autoregulation (CA) [1]–[3], has not yet been explored in the neurosurgical setting, despite its potential relevance. First of all, patients with disturbed CA during other surgical procedures have shown higher risk of perioperative stroke [4], acute kidney injury [5] or delirium [6]. Secondly, periods of hypotension might occur during the neurosurgery [7]. As arterial blood pressure (ABP) targets management frequently relies on a ‘one size fits all’ strategy (following standard recommendations [8]), this may result in ABP dropping below the individual lower limit of autoregulation (LLA) potentially causing cerebral ischaemia [9]. Lastly, non-invasive near-infrared spectroscopy (NIRS) derived cerebral regional oxygen saturation (rSO_2_) is available and commonly used during neurosurgical operations. Slow fluctuations (20 s to 3 min) of rSO_2_ can be considered as a surrogate measure of slow waves of cerebral blood flow (CBF) [10], providing an index of CA (COx), a correlation coefficient between vasogenic changes in rSO_2_ and ABP [11]. When plotted against ABP over a period of hours, COx often reveals a U-shape curve characteristic. The minimum (i.e. optimal point) of the curve identifies the ABP value at which CA is best preserved. We name this value ‘optimal ABP’ (ABP_OPT_). For ABP lower than ABP_OPT_ (left side of the U-shape curve), and for high levels of COx denoting impaired CA, we can identify values of ABP at the LLA.

We aim to assess performance of a modified algorithm for continuous, automated calculation of COx-derived ABP_OPT_ and LLA in patients undergoing elective neurosurgery. Our group has pioneered the technology used for continuous estimation of ‘optimal’ cerebral perfusion pressure in the realm of traumatic brain injury patients admitted in intensive care unit (ICU) [2, 12, 13]. The challenge we face in the neurosurgical setting is represented by the fact that the duration of the surgery (and therefore the monitoring time) is shorter when compared to the length of the ICU monitoring time. Hence, the technology used in ICU cannot be directly translated into the operating room.

As a secondary objective, we aim to explore the differences between the time trends of ABP_OPT_ and LLA compared with the preoperative and intraoperative ABP.

## Methods

This is an observational study. We performed a retrospective analysis of prospectively collected data. This manuscript adheres to the applicable STROBE guidelines (Appendix 3).

### Study approval

The Hospital Clinic de Barcelona Institutional Research Ethics Committee (CEIm HCB/2018/1173) approved this research. Written informed consent was waived by the Ethics Committee.

### Patients

Consecutive adult patients undergoing elective brain and spinal surgery were enrolled from October 2015 to September 2018 at the Hospital Clínic de Barcelona. Patients were considered eligible if the operations were expected to last at least 2 h and if the anaesthetic management required continuous invasive ABP and non-invasive rSO_2_ monitoring (Covidien INVOS 5100C Device, Covidien Company USA) according to the local clinical practice. Intraoperative magnetic resonance imaging was an exclusion criterion as it requires specific compatible monitoring devices.

All patients underwent total intravenous anesthesia with target-controlled infusion modalities, using propofol and remifentanil. Rocuronium was administered as a bolus to facilitate orotracheal intubation in all patients. Continuous infusion was administered only for cases where continuous intraoperative neuromonitoring (electromyography, motor evoked potentials) was not required.

Normocapnia was maintained throughout the surgeries according to end-tidal carbon dioxide (EtCO_2_) and arterial blood gases monitoring. All patients were managed according to the locallly established practice which aimed to keep the intraoperative mean ABP as close as possible to baseline individual preoperative values of mean ABP (‘ABP_BASELINE_’, see data collection subsection).

### Data collection

Baseline mean ABP (ABP_BASELINE_) was calculated as the average of three non-invasive pre-operative ABP measurements conducted at the admission ward [14].

Full waveform resolution digital output of ABP and end-tidal carbon dioxide (EtCO_2_) from the vital signs monitor (CARESCAPE B850, General Electric) and the regional saturation of oxygen (rSO_2_) from the NIRS monitor were streamed in real-time into the ICM + software [15] [https://icmplus.neurosurg.cam.ac.uk] running on a laptop computer. All the data were synchronised and integrated at 100 Hz sampling frequency.

The recording session in ICM + started as soon as the arterial line was inserted and before induction when possible. The following information was retrieved from each patient’s medical record: age, sex, type and position of surgery, rocuronium infusion (NMBA).

### Data processing

ICM + software was used for all data pre-processing of individual high-resolution recordings prior to statistical analysis.

The data from all recording sessions were assessed visually by the authors and the signals were classified according to their quality. Recordings with low or unstable amplitude of ABP signal, unreliable values of ABP and rSO_2_ signal and/or non availability of ABP or rSO_2_ signals were classified as ‘poor quality’ data and excluded. Only recordings with EtCO_2_ were included (to ensure normocapnia). The artifacts in ABP (arterial line flushing, errors of measurement) were removed manually or automatically (via pulse detection method). rSO_2_ values < 20% were excluded as artifact. When rSO_2_ was measured bilaterally, the right side was selected for analysis because most unilateral recordings were right-sided. ABP and rSO_2_ recorded waveforms were down-sampled to 0.1 Hz by coarse-graining using 10 s, non-overlapping averages.

**Fig. 1:**
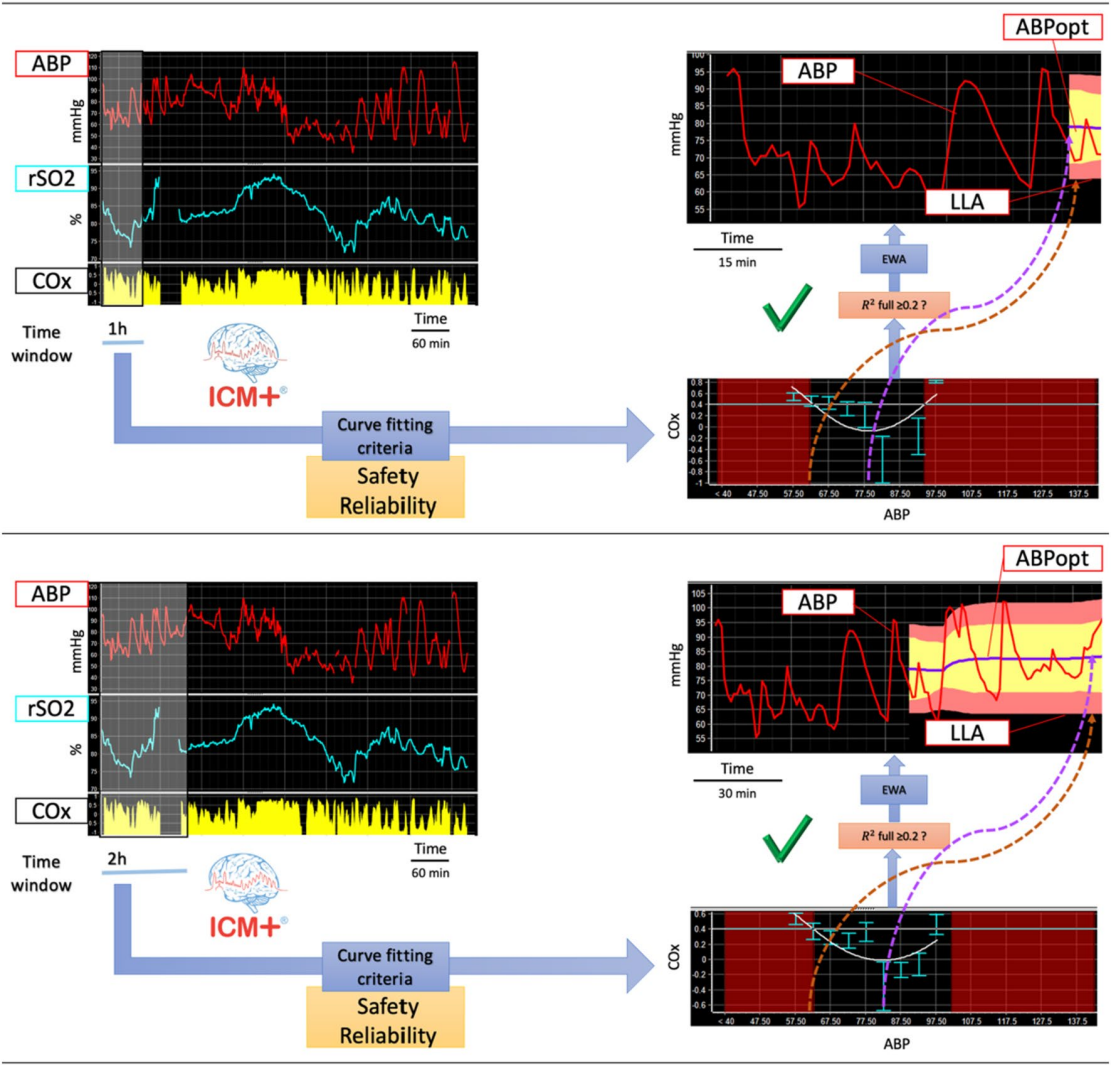
Expanding window approach for assessment of ABP_OPT_ and LLA time trends. After the first h of data (upper panel), ABP and COx values are used for plotting COx-ABP relationship according to curve fitting criteria selected for their performance in safety and reliability. If the quality control check is fulfilled, then smoothing is applied (EWA) and ABP_OPT_ and LLA values are estimated. The whole process is updated every minute, resulting in ABP_OPT_ and LLA time trends. The lower panel shows how the process continued up to 2 h. *ABP* arterial blood pressure, *rSO*_*2*_ regional cerebral saturation of oxygen, *COx* cerebral oximetry index, *EWA* exponentially weighted average, *ABP*_*OPT*_ optimal blood pressure, *LLA* lower limit of autoregulation

Cerebral oximetry index (COx) was calculated as a moving Pearson correlation coefficient between 30 consecutive, 10-s averaged values of ABP and rSO_2_, updated every minute[11]. COx values were Fisher-transformed prior to any statistical analysis[16]. ABP_OPT_ and LLA time trends were derived using a single expanding window, anchored at the start of recording, specifically designed for this project and explained in detail in Appendix 1 and Fig. 1*.* Briefly, in this new algorithm, after the first hour of data, all the previous ABP and COx values (including the data from the first hour) are used for plotting COx-ABP relationship according to curve fitting criteria selected for their performance in safety and reliability. A quality control check determines whether ABP_OPT_ and LLA values can be estimated. The whole process is updated every minute, resulting in ABP_OPT_ and LLA time trends.

ABP, rSO_2_, COx and LLA values referred to in this study are 60-s means.

### Statistical analysis

Statistical analysis was performed with R statistical language v4.0 [17]. Normality of continuous variables was assessed with histograms, quantile–quantile plots and Shapiro–Wilks test.

The assumption of normocapia and stability in EtCO_2_ was verified with visual inspection of the time trends of EtCO_2_, and evaluation of the standard deviation of EtCO_2_ during the recordings (named “EtCO_2_ variability”).

#### Primary objective

For the primary objective we determined: availability (number of patients with ABP_OPT_ or LLA available); time to achieve ABP_OPT_ and LLA (calculated as time required for the automated algorithm to generate the first calculated value).

#### Secondary objective

The difference in median values between ABP_OPT_ and ABP_BASELINE_ was assessed using the Mann-U test.

The relationship between ABP_OPT_ and ABP was explored with different approaches, listed below. A significance level of 0.05 was considered, unless otherwise specified.

1. Average total amount of ABP above and below ABP_OPT_ (delta), dose (mmHg*hours) and % of ABP recorded time with ABP above or below ABP_OPT_ (± 5 mmHg) were calculated for the whole recorded period and averaged for the whole cohort of patients. This approach however does not take into account interpatient variability.
2. Within patient correlation of min-by-min ABP_OPT_ and ABP values was explored with ABP_OPT_-ABP scatterplot and Pearson correlation first. However, this approach does not take into account autocorrelation between samples of each variable. Hence we modeled ABP_OPT_ and ABP as time series (see Appendix 2).
3. We assessed bidirectional Granger causality test (GC) [18] for each patient to examine whether the variability in ABP_OPT_ time series could be directly explained by the variability in ABP time series. GC was performed on differentiated ABP_OPT_ and ABP time series (calculated as the first differential of the time series and referred to as ABPopt.diff and ABP.diff in this manuscript) with lag order set at 1 in order to address the stationarity assumption of this analysis method. Significance level for the GC was set at 0.025 accounting for two tests performed for each patient (ABP.diff → ABPopt.diff and ABPopt.diff → ABP.diff)
4. We created linear mixed effect (LME) models to investigate whether for each patient ABP_OPT_ and ABP time trends were linearly correlated. Fixed effects were ‘ABPopt.diff,’ ‘ABP.diff’ and ‘Time.’ Random effects were ‘Patient’ and ‘Time’. Models with and without the correlation structure ARMA (1,1) were considered.
5. The cohort time profile of ABP and the cohort time trend of the estimation of ABP_OPT_ were explored with generalized additive method (GAM) smoothing with cubic spline. Time was anchored to the beginning of the recording. We explored GAM models applied to groups of patients defined according to different duration of their recordings, which relates to the duration (and type) of surgery. We identified a breaking point at 5 h: recordings shorter than 5 h had a similar time profile pattern between them, and recordings longer than 5 h were similar between them. Hence, we present the GAM models for patients with recordings lasting less than 5 h (short duration) separately from patients with recordings lasting between 5 and 10 h (long duration). Although this approach looks at cohort-based time variability, it allows capturing important patterns in the data.

The relationship between LLA and ABP was investigated in terms of number of episodes of ABP below LLA, which we named ‘hypotensive insults’. We did not assume, nor had data to proof, the fact that these ‘insults’ were or were not associated with clinically relevant ischemic events. Each insult was defined as an event of ABP dropping below LLA for at least 60 s. We assessed also average and maximum dose and % of time with ABP below LLA.

## Results

A total number of 99 patients were initially enrolled. Fourteen patients were excluded at the analytical phase due to: poor quality of data (n = 10); absence of EtCO_2_ (n = 3); length of continuous data with both ABP and rSO_2_ shorter than 60 min (n = 1, see Appendix 1 for details).

Table 1 shows demographic and baseline characteristics of the 85 patients included. The patients were on average 60 years old and 55% were females. The most common type of surgery was supratentorial surgery (~ 33%), followed by infratentorial surgery (~ 21%). 65% of patients were in the supine position during their operation.

**Table 1:** Demographic and baseline characteristics of patients included in the analysis

Variable	N or Median	% or IQR
Age		
Age (years)	60	(51–68)
Sex		
F	47	55.3
M	38	44.7
Surgery type		
Epilepsy	11	12.9
Functional	2	2.4
Infratentorial	18	21.2
Skull base	2	2.4
Spine	9	10.6
Supratentorial	28	32.9
Vascular	15	17.6
Position		
Lateral	10	11.8
Prone	13	15.3
Seated	7	8.2
Supine	55	64.7
NMBA		
No	47	55.3
Yes	38	44.7
Duration of recordings		
Duration (min)	287	(211–365)

*NMBA* neuromuscular blocking agents, *IQR* interquartile range

Table 2 shows descriptive statistics for main monitoring variables, primary objective endpoints results, and main results for ABP_OPT_-ABP and LLA-ABP relationships analysis. In both tables, descriptive data are expressed as median (IQR). EtCO_2_ was considered stable in all the 85 recordings. EtCO_2_ variability spanned from 0.23 to 0.38 with a highest value of 0.88 (not shown in the table). Hence all recordings were considered suitable for the anbalysis. On average COx values spanned from negative (1st quartile − 0.12) to positive (3rd quartile 0.43) values.

**Table 2:** Descriptive statistics for main monitoring variables, primary objective endpoints results and main results for analysis of the relationship ABP_OPT_-ABP and LLA-ABP for the 85 patients included in the analysis

Variable	N or Median	% or IQR
Monitoring variables		
ABP (mmHg)	76.48	(71.37–81.05)
ABP_BASELINE_ (mmHg)	90	(83–95)
EtCO_2_ (KPa)	4.13	(3.90–4.35)
EtCO_2_ variability (KPa)	0.31	(0.23–0.38)
rSO_2_ (%)	71.62	(64.2–78.3)
COx	0.1259	(− 0.1181–0.4047)
COx (after Fisher transformation)	0.1266	(− 0.1187–0.4293)
Availability		
ABP_OPT_	73	86
LLA	76	89
Time to achieve the first value (min)		
ABP_OPT_	97	(79–155)
LLA	92.5	(77.5–122.3)
Time to achieve the first value relative to the total duration (%)		
ABP_OPT_	38.4	(24.5–53.9)
LLA	39.65	(25.0–61.8)
Relationship ABP_OPT_-ABP_REAL_^(*)^		
Delta above ABP_OPT_ (mmHg)	8	(5–12)
Patients with Delta above ABP_OPT_ > 5 mmHg	54	74
Delta below ABP_OPT_ (mmHg)	5	(1–10)
Patients with Delta below ABP_OPT_ > 5 mmHg	39	53
Dose above or below ABP_OPT_ ± 5 mmHg (mmHg*h)	7	(4–12)
Time above or below ABP_OPT_ ± 5 mmHg (%)	71.87	(55.63–85.71)
Relationship LLA-ABP		
Patients with insults below LLA	32	38

(*) Delta ABP below or above ABP_OPT_ are presented as absolute values. Delta above ABP_OPT_ was calculated as ABP–ABP_OPT_ provided that ABP > ABPopt. Delta below ABP_OPT_ was calculated as ABP_OPT_ – ABP provided that ABP < ABPopt. For the counts, a margin of 5 mmHg around ABP_OPT_ was used

*ABP* intraoperative arterial blood pressure, *ABP*_*BASELINE*_ baseline preoperative values of mean arterial blood pressure, *EtCO*_*2*_ end tidal CO_2_, *COx* cerebral oximetry index, *ABP*_*OPT*_ optimal arterial blood pressure, *LLA* lower limit of autoregulation, *IQR* interquartile range

In 12 patients, ABP_OPT_ and LLA were not available. The reason for this non availability is either that there was not enough variability in blood pressure to ‘probe’ the autoregulatory curve, or that there was insufficient variability in COx across the range of recorded ABP values (indicating either uniformly disturbed CA, or uniformly preserved CA, or flat CA curve in that range of ABP values). We noticed that in those patients there was a relatively high prevalence of prone position (33% in patients without ABP_OPT_ as opposed to 15% in the whole cohort).

**Fig. 2:**
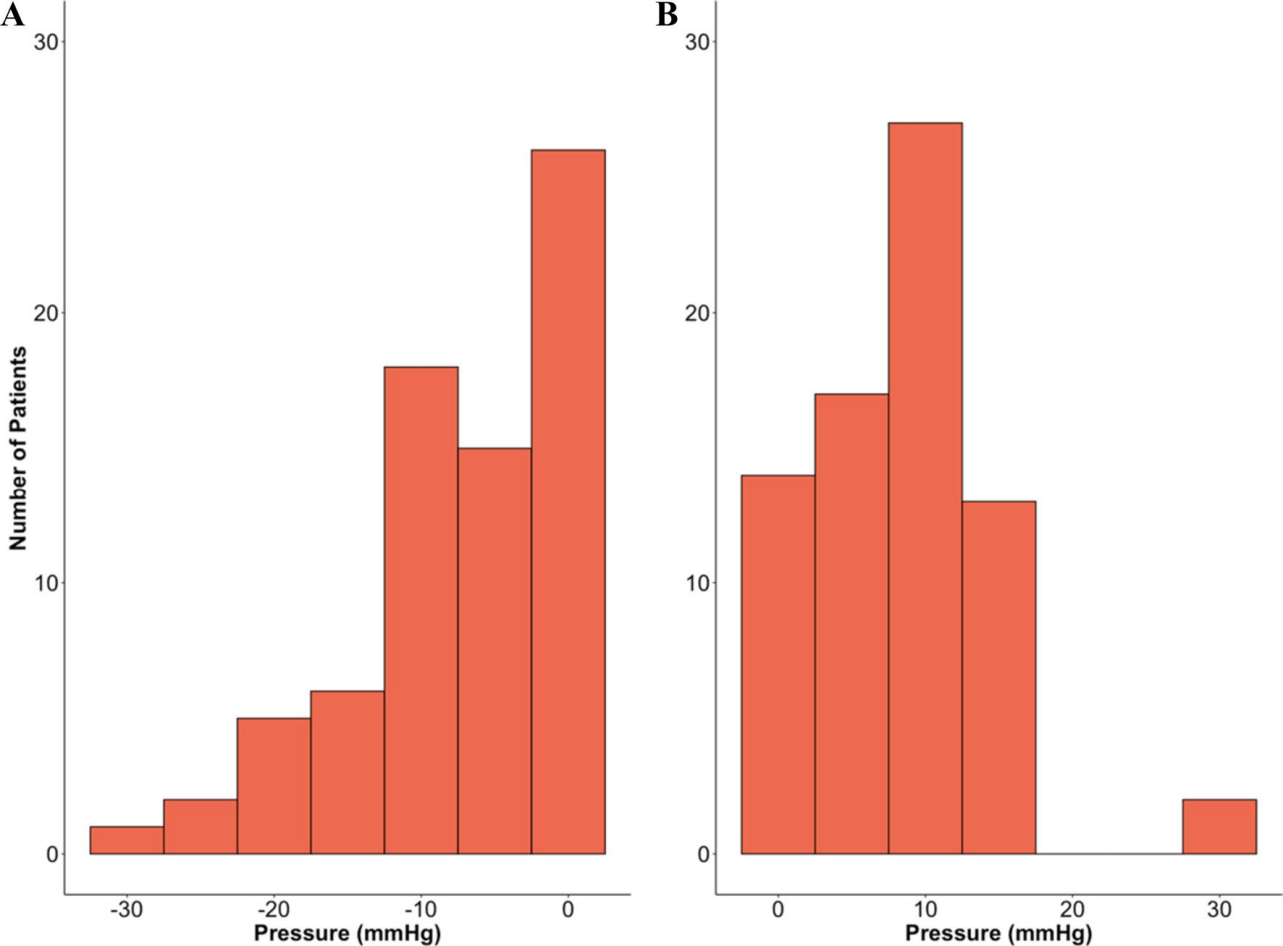
Distribution of average delta ABP below and above ABP_OPT_. Deviation of arterial blood pressure (ABP) from the autoregulation based optimal blood pressure (ABP_OPT_) was assessed for both periods when ABP was below (**A**) and above (**B**) ABP_OPT_

Figure 2 represents the distribution of average deviation (or delta) of ABP above or below ABP_OPT_. In 54 (74%) patients with ABP_OPT_ available, delta ABP above ABP_OPT_ was higher than 5 mmHg. For 39 (53%) patients, delta ABP below ABP_OPT_ was greater than 5 mmHg.

**Fig. 3:**
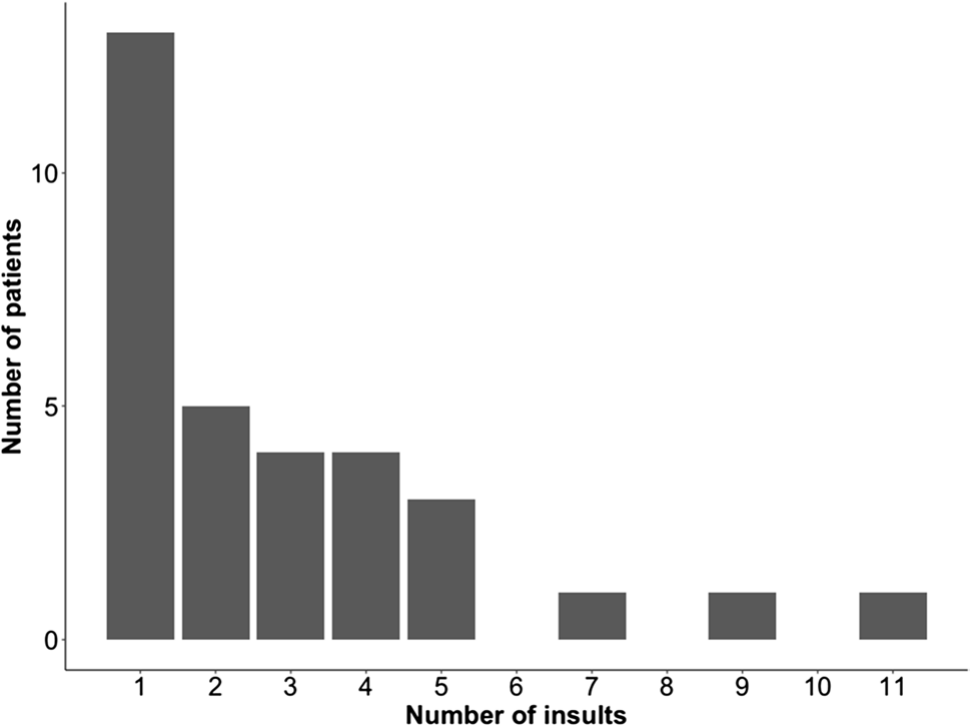
Distribution of occurrence of hypotensive events of ABP below LLA. Distribution of occurrence of hypotensive events with ABP below LLA for at least 60 s among the 32 patients that presented these events. *ABP* intraoperative arterial blood pressure, *LLA* lower limit of autoregulation

Median(IQR) ABP_OPT_ (75 mmHg (69–84)) was different from ABP_BASELINE_ [90 mmHg (84–95)] (*p* < 0.001, Mann-U test), suggesting that on average ABP_OPT_ was 15 mmHg lower than ABP_BASELINE_. Median (IQR) dose of ABP below LLA was 0 (0–0.2), but maximum value in one patient was 19 mmHg*h. Similarly, median time spent with ABP below LLA was 0 (0–6)%, but maximum value was 94%. The distribution of occurrence of hypotensive insults with ABP below LLA in the 32 patients that had such insults (38%) is shown in Fig. 3.

Scatterplots and Pearson correlation coefficients of min-by-min ABP_OPT_ and ABP values were examined for each patient (not reported here). Overall, we did not identify any particular pattern that would describe the correlation between ABP_OPT_ values and ABP values in individual patients.

Granger causality test was non significant (*p* > 0.025) for 70/73 tests with direction ABP.diff → ABPopt.diff and for 64/73 tests with direction ABPopt.diff → ABP.diff. This supports the hypothesis that the variability in ABP_OPT_ time series is not simply mathematically related to the variability in ABP time series.

Linear mixed effect models were built to investigate whether ABP_OPT_ time trends were independent from ABP time trends within patients. None of the models showed any significance for any of the explanatory variables, even after model reduction. In particular we observed that the correlation structure was needed but none of these contributions were significant: ABP.diff, time, time as fixed effect, the interaction term ABP.diff:Time.

These results seem to suggest that ABP_OPT_ variability did not exhibit a linear time trend and it was not dependent on ABP time trend within patients.

**Fig. 4:**
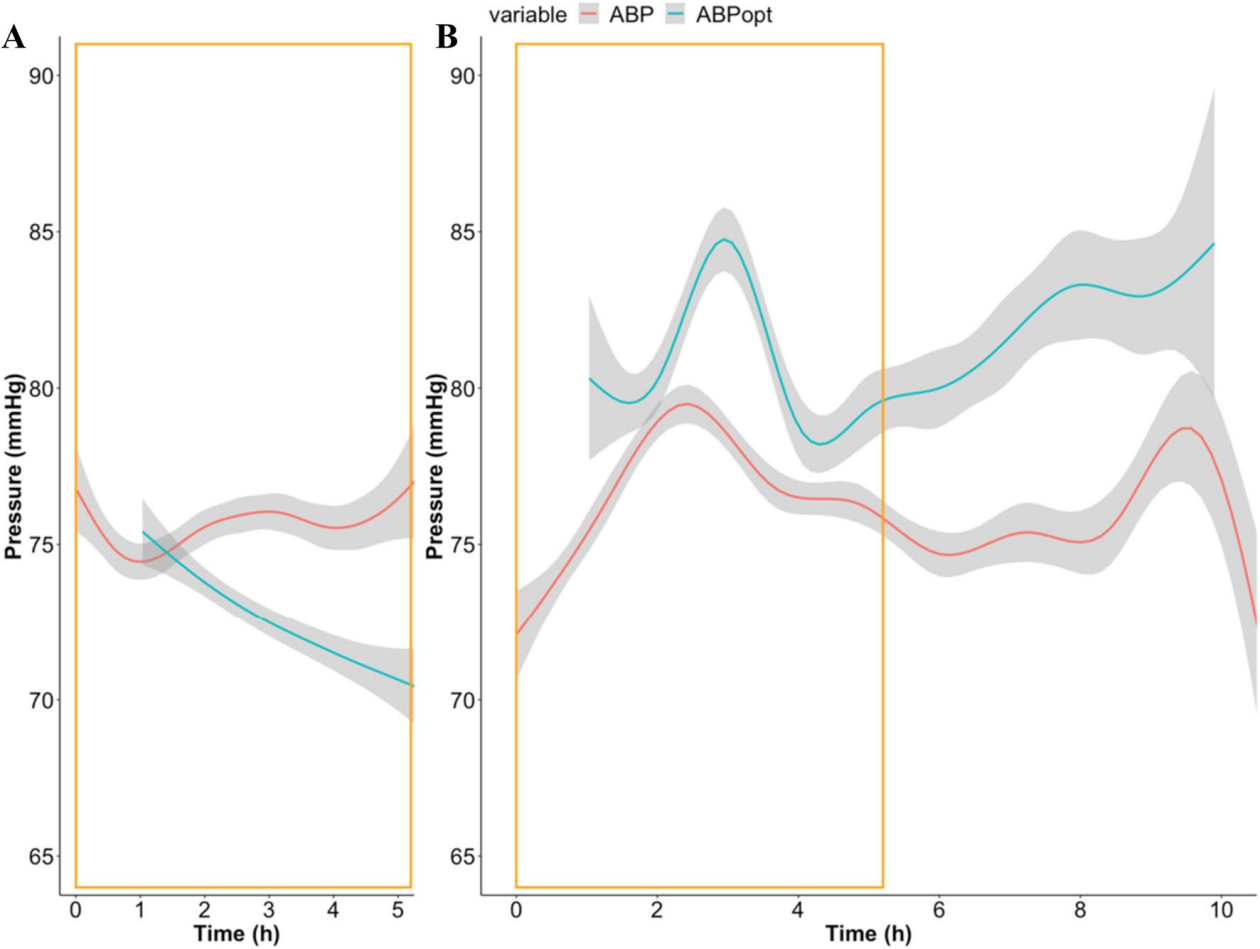
Time profile of measured ABP and time trend of the estimation of ABP_OPT_ for two surgery duration groups. ABP time profile (here named ABP) and the time trend of the estimation of ABP_OPT_ are represented for patients with **A** recordings lasting less than 5 h (corresponding to shorter surgeries, n = 41) and **B** recordings lasting from 5 to 10 h (corresponding to longer surgeries, n = 29). Smoothing was achieved with generalized additive model (GAM) using cubic spline. *ABP* arterial blood pressure, *ABP*_*OPT*_ optimal arterial blood pressure

Figure 4 shows the time profile of ABP values and of the estimation of ABP_OPT_ for the whole cohort split into two groups, according to the length of surgery.

## Discussion

Our study presents evidence for the assessment of continuous individualised automated arterial blood pressure (ABP) targets for patients undergoing elective neurosurgery. Targeting intraoperative ABP at the estimated ‘optimal value’ ABP_OPT_ or above lower limit of autoregulation (LLA) could provide means for preserving cerebral autoregulation protective mechanism. This could potentially translate to reduced occurrence of postoperative poor outcomes.

We first proposed a method for determining continuous COx-based ABP_OPT_ and LLA in neurosurgical settings. We revised the technology developed for the realm of traumatic brain injury in ICU [2, 19, 20] by introducing an expanding window approach (see Appendix 1 and Fig. 1). We were able to compute ABP_OPT_ and LLA time trends retrospectively in majority of the patients, more than 85% and almost 90% patients respectively. In patients without ABP_OPT_ (n = 12) there was a relative high prevalence of prone positioning and the variability in ABP and rSO_2_ signals in those recordings was very poor. Although we could not find a plausible explanation for this effect it must be stressed that variability in ABP is a perquisite for autoregulation estimation [21–23]. In that case, the automated algorithm had rejected U-shape curves that would not satisfy the quality control criteria (see data processing methods). While this might give the impression of a limitation for the feasibility of this method, we believe that it represents a feature of strength in terms of safety and reliability. The expanding window algorithm can be implemented in the ICM + software to use with intraoperative real-time high-resolution data collection, making ABP_OPT_ and LLA time trends available for the anaesthesiologist (see Fig. 1).

Duration of elective neurosurgeries is much shorter than the average ICU length of stay. As a consequence, the time required to achieve the first reliable value of Optimal ABP and LLA targets is particularly relevant here. In our exploratory analysis we could obtain the first ABP_OPT_ and LLA values just after an hour and a half of recording. We compared this time lag to the total duration of the recording (close to the total duration of the surgery) and found that both targets could be available for more than 60% of the monitoring time. This supports the feasibility of such approach. Of note, preparation of patients in elective neurosurgery takes longer than many other surgical specialties [24]. Hence, we speculate that NIRS and ABP monitoring could be initiated early enough to provide an acceptable buffer of data that would allow a reliable evaluation of ABP_OPT_ and LLA available for the beginning of surgery. However, we could not investigate this hypothesis due to the limited number of patients that had arterial line pre intubation in our cohort.

Preoperative arterial blood pressure is often considered when adjusting the intraoperative arterial blood pressure target. We calculated ABP_BASELINE_ as the mean of ABP measures in the hours previous to the operation and demonstrated that ABP_OPT_ is on average lower than ABP_BASELINE_. This suggests that targeting the preoperative ABP might not be the optimal choice, from the point of view of preservation of cerebral autoregulation in patients undergoing neurosurgery, while on the other hand risking subjecting the patient to overly, unnecessarily, high ABP. The latter has been suggested to be associated with postoperative delirium in cardiac surgery patients [25] and with an increased risk of seizures in SAH patients [26]. We did not however investigate the upper limit of autoregulation (ULA) in our cohort, given that the assessment of ULA has not yet been validated experimentally, and that the notion of a ULA may be a lot more elusive, given the recent evidence [27]. Nevertheless the negative effects of unneeded high ABP certainly needs further attention. In this cohort, the intraoperative CA status fluctuated over a short period of time (as shown by high variability in COx) and patients spent more than 70% of the ABP monitored time with their intraoperative ABP above or below ABP_OPT_ ± 5 mmHg (Table 2). Whether the time spent with ABP far from ABP_OPT_ is clinically relevant remains to be established, as we do not have postoperative outcomes for this cohort. However, these findings seem to support the necessity of monitoring CA to be unblinded with respect to cerebral perfusion. To reinforce this hypothesis, we showed that we could detect possible hypotensive events, which could potentially lead to cerebral hypoperfusion, defined with ABP dropping below the calculated LLA time trend. Almost 40% of the patients in our cohort had at least one such episode (Table 2). A few patients had more than five hypotensive insults (Fig. 3). Intraoperative hypotension has been associated to postoperative delirium or renal failure [6]. Hypotensive insults detected in our cohort might have been driven by the vasodilatory effect of the anesthetic drugs, or might have occurred in an unnoticed way, simply because an individualised and dynamic LLA time trend was not available for the anaesthetist. Further investigations are required to validate the association of our findings with postoperative outcomes.

Is our estimation of Optimal ABP truly different from measured arterial blood pressure? ABP_OPT_ is calculated and estimated from values of ABP according to the COx/ABP relationship (see data processing methods). If this mathematical relationship resulted in ABP_OPT_ values being simply linearly related to ABP, then one might argue that assessing ABP_OPT_ would be of little usefulness. We explored this matter tackling different angles. We investigated whether the variability of one time series could be explained by variability of the other using bidirectional Granger Causality method and showed that this was not the case for majority of patients (because Granger Causality is a statistical tool, we expect some tests to be significant by chance). Further, we looked at linear mixed effect models which take into account the time trend and the within patients variability. We were not able to model ABP_OPT_ time series as if it was related to ABP within patients. Finally, we explored the time profiles of ABP and ABP_OPT_ (Fig. 4). We observed that the time profile behaves differently between long and short surgeries. We do not know whether this difference had an underlying clinical explanation. Certainly, this aspect requires more investigation. We speculate that this might translate into different application of the concept of individualising ABP targets according to CA depending on the duration or type of surgery. Although here we look at cohort trends, rather than individual patients’ trends, we could appreciate that ABP_OPT_ and ABP time trends were different. Our results agree with the hypothesis that ABP_OPT_ is independent from ABP within patients. This supports the usefulness of continuous monitoring of ABP_OPT_.

### Limitations

Our study has several limitations. First of all, this pilot study lacks clinical outcome measurements. Hence, it is not feasible to draw any practical clinical benefit from our results at this point. Second, the position of NIRS probes depended on the neurosurgical approach. Most of our cases had only unilateral measurement, located at the opposite side of the craniotomy. In most of the patients’ recordings and for majority of time, the cranial vault was open. Whether this fact influences our results and interpretations remains to be confirmed [28]. Third, cerebral perfusion pressure (CPP) could not be estimated, because intracranial pressure (ICP) was not measured and central venous pressure (CVP) was not available. We assumed that CVP and ICP were constant or negligible in our patients. Hence, we considered ABP as a surrogate for CPP. Fourth, the sample size was a matter of convenience. To date, there are no studies available on the subject that could guide our sample size calculation. We aimed to address our questions in our retrospective dataset and with this sample size we were able to find answers to our objectives.

## Conclusion

We were able to assess, retrospectively, autoregulation-based individualised automated ABP targets in the neurosurgical setting. This could translate in real-time intraoperative clinical application that might reduce peri-operative complications. Further investigation is required to assess its relationship with postoperative outcomes.

## Fundings

Erta Beqiri is supported by the Medical Research Council (Grant No.: MR N013433-1) and by the Gates Cambridge Scholarship. Frederick A. Zeiler is supported through the Manitoba Public Insurance (MPI) Professorship in Neuroscience/TBI Research Endowment, the Natural Sciences and Engineering Research Council of Canada (NSERC; DGECR-2022-00260, RGPIN-20220-3621, ALLRP-576386-22, ALLRP-578524-22), Canadian Institutes of Health Research (CIHR), the MPI Neuroscience Research Operating Fund, the Health Sciences Centre Foundation Winnipeg, the Canada Foundation for Innovation (CFI) (Project #: 38,583), Research Manitoba (Grant #: 3906 and 5429) and the University of Manitoba VPRI Research Investment Fund (RIF). Marcel Aries and Jeanette Tas are supported by a grant from the ‘Brain Battle’ Foundation (HersenStrijd fonds) from the University Maastricht, The Netherlands. Marek Czosnyka is supported by National Institute for Health Research (NIHR), Cambridge Biomedical Research Centre.

## Appendix 1—Expanding window approach for assessment of ABP_OPT_ and LLA

Firstly, the cerebral oximetry index (COx) is Fisher-transformed to ensure its normality and avoid bias for optimal blood pressure (ABP_OPT_) calculations [16]. All the data points are subsequently divided into groups corresponding to intraoperative arterial blood pressure (ABP) bins of 5 mmHg, within 40–140 mmHg of ABP values. For each bin, mean COx and ABP values are used to fit a 2nd order polynomial describing the theoretical U-shape curve. At each time point, a COx-ABP plot is generated from the past data (using a particular buffer as explained below) according to curve fitting criteria adapted from previously published methods [2, 19] as follows:

1. Each ABP bin must represent at least 3% of the total data count: ABP values that are very scarcely represented, likely due to short spikes or drops, but not to the physiological trend, will be disregarded.
2. At least 50% of the data in the time window must be included in the curve fit.
3. COx variation of at least 0.2 is mandated (thus rejecting flatter COx-ABP curves).
4. The COx range of interest is enforced to be between 0 and 0.7, so the algorithm will not return any value when COx is always very high (indicating a complete loss of blood flow regulation) or always very low (autoregulation preserved at each ABP value).
5. The coefficient of determination of the fitted curve R^2^ (calculated also for the bins excluded from the curve fitting process) must be at least 0.2.

Assuming that the event ‘neurosurgery’ does not affect the patient’s cerebral autoregulation (CA), calculations of ABP_OPT_ and lower limit of autoregulation (LLA) can be performed on an expanding data buffer (window) with, in theory, ever increasing accuracy. Preliminary exploration of the data from a randomly selected group of patients by visual inspection of the Cox-ABP plots, revealed that the minimum time required to have a reliable curve was between 45 and 75 min. Thus, as a starting point for the expanding calculation window algorithm, a duration of data buffer of 1 h (that is 60 data points) was chosen. All the calculations leading up to ABP_OPT_ and LLA (as detailed below) were performed on this buffer, and on subsequently, iteratively, enlarged buffers.

If the curve fulfills the quality control as assessed with criterion 5, then is accepted and the function returns an output resulting in a time series which is finally subjected to an exponentially weighted average (EWA) filter of 10 min of duration, forming the final time trend. The EWA weight is calculated as ${\left(1- a\right)}^{{\text{k}}}$ where *k* is the distance, in number of samples, from the current sample and $a$ is set at 0.1. This process is repeated for each progressively longer data window. When the output is the optimum of the fitted curve, then we name it ABP_OPT_. Only when the curve is parabolic (U-shaped, but not ascending or descending) the value is accepted and the result is the ABP_OPT_ time trend. When the output is ABP corresponding to a certain threshold of Cox at which autoregulation is lost, we name it LLA (left side of the curve). We explored different thresholds of COx for defining lost autoregulation. Since the results of the statistical analysis were qualitatively identical, we report only results for LLA at COx = 0.4, which represents a level of severely impaired autoregulation. In this case, we were interested in the left side of the curve. Therefore, when a parabolic curve was not available, the non-parabolic descending curve was accepted for the LLA assessment.

Figure 1 provides a schematic representation of the algorithm.

## Appendix 2—Time series modelling

For Granger causality test and linear mixed effect models we adopted time series modelling techniques (Auto Regressive Integrated Moving Average—ARIMA) as follows.

- *Data selection*: The time period considered for each patient started from the first available value of ABP_OPT_ and ended at the last available value of ABP_OPT_. Gaps were filled in with the last available value before the gap (as this would be clinically meaningful in real-time application of the concept).
- *Stationarity*: Stationarity of each time series was assessed with autocorrelation function (ACF) correlogram, Kwiatkowski–Phillips–Schmidt–Shin (KPSS) test and Augmented Dickey–Fuller (ADF) test. Both time series were non stationary for majority of the patients, therefore they were differentiated (differencing order d = 1) regardless if they initially violated the assumption of stationarity or not[29]. After differentiation, ACF plots, ADF and KPSS tests were re-run, confirming reduction of trend in the ACF plots and lack or significant root or higher order trend seen on ADF/KPSS testing. Differentiated ABP_OPT_ and ABP time series will be refered to as ABPopt.diff and ABP.diff in this manuscript.
- *Autoregressive and moving average structure*: Initial estimate of the autoregressive structure (*p*) of ABPopt.diff and ABP.diff was inferred from partial autocorrelation (PACF) correlograms of individual time series. Similarly, moving average component (*q*) of ABPopt.diff was inferred from ACF correlograms. Subsequently we ran sequential ARIMA models (*p,d,q*) for ABPopt.diff by varying the order of *p* and *q* creating different combinations starting from their initially estimated values. The differencing order *d* was fixed at 1, as we used differentiated time series at this stage and no further differentiation was required. Akaike information criterion (AIC) and Bayesian information criterion (BIC) were used to estimate the best common ARIMA model for ABP_OPT_, based on the principle of parsimony. This led to the final optimal model of (1,1,1). All these estimations were performed in ten randomly selected patients, chosen to represent different duration of recordings and duration of gaps.
- *Granger causality*: Granger causality was used to test causal relations between ABPopt.diff and ABP.diff. Lag order was set at 1, as suggested by the autoregressive structure of both ABP_OPT_ and ABP. Bonferroni-adjusted significance level was set at 0.025 accounting for two tests performed for each patient (ABP.diff → ABPopt.diff and ABPopt.diff → ABP.diff causality).
- *Linear mixed effect models*: ABPopt.diff was considered the response variable in linear mixed effect (LME) models. The correlation structure was defined as ARMA order (1,1). Note that ARMA (1,1) for differentiated time series is equivalent to ARIMA (1,1,1) for non differentiated time series, as ARIMA modelling of errors is not available in R package ‘nlme’ used for this analysis. Fixed effects considered for LME models were ‘ABPopt.diff,’ ‘ABP.diff’ and ‘Time.’ Random effects were ‘Patient’ and ‘Time.’ Models with and without the correlation structure were considered. Model reduction was explored.

## Appendix 3—STROBE statement checklist

Here we report the STROBE statement checklist for observational studies as applied for our study. Where appropriate, the item of the checklist has been marked with “X”, indicating that our study followed the recommendation.

**Table:**

	Item No	Recommendation
Title and abstract	1	**X** (*a*) Indicate the study’s design with a commonly used term in the title or the abstract
		(*b*) Provide in the abstract an informative and balanced summary of what was done and what was found
Introduction		
Background/rationale	2	**X** Explain the scientific background and rationale for the investigation being reported
Objectives	3	**X** State specific objectives, including any prespecified hypotheses
Methods		
Study design	4	**X** Present key elements of study design early in the paper
Setting	5	**X** Describe the setting, locations, and relevant dates, including periods of recruitment, exposure, follow-up, and data collection
Participants	6	(*a*) **X** *Cohort study*—Give the eligibility criteria, and the sources and methods of selection of participants. Describe methods of follow-up *Case–control study*—Give the eligibility criteria, and the sources and methods of case ascertainment and control selection. Give the rationale for the choice of cases and controls *Cross-sectional study*—Give the eligibility criteria, and the sources and methods of selection of participants
		(*b*) *Cohort study*—For matched studies, give matching criteria and number of exposed and unexposed *Case–control study*—For matched studies, give matching criteria and the number of controls per case
Variables	7	**X** Clearly define all outcomes, exposures, predictors, potential confounders, and effect modifiers. Give diagnostic criteria, if applicable
Data sources/measurement	8	**X** For each variable of interest, give sources of data and details of methods of assessment (measurement). Describe comparability of assessment methods if there is more than one group
Bias	9	Describe any efforts to address potential sources of bias
Study size	10	Explain how the study size was arrived at
Quantitative variables	11	**X** Explain how quantitative variables were handled in the analyses. If applicable, describe which groupings were chosen and why
Statistical methods	12	**X** (*a*) Describe all statistical methods, including those used to control for confounding
		**X** (*b*) Describe any methods used to examine subgroups and interactions
		**X** (*c*) Explain how missing data were addressed
		(*d*) *Cohort study*—If applicable, explain how loss to follow-up was addressed *Case–control study*—If applicable, explain how matching of cases and controls was addressed *Cross-sectional study*—If applicable, describe analytical methods taking account of sampling strategy
		(*e*) Describe any sensitivity analyses
Results		
Participants	13	**X** (a) Report numbers of individuals at each stage of study—eg numbers potentially eligible, examined for eligibility, confirmed eligible, included in the study, completing follow-up, and analysed
		**X** (b) Give reasons for non-participation at each stage
		**X** (c) Consider use of a flow diagram
Descriptive data	14	**X** (a) Give characteristics of study participants (eg demographic, clinical, social) and information on exposures and potential confounders
		(b) Indicate number of participants with missing data for each variable of interest
		(c) *Cohort study*—Summarise follow-up time (eg, average and total amount)
Outcome data	15	*Cohort study*—Report numbers of outcome events or summary measures over time
		*Case–control study—*Report numbers in each exposure category, or summary measures of exposure
		*Cross-sectional study—*Report numbers of outcome events or summary measures
Main results	16	**X** (*a*) Give unadjusted estimates and, if applicable, confounder-adjusted estimates and their precision (eg, 95% confidence interval). Make clear which confounders were adjusted for and why they were included
		**X** (*b*) Report category boundaries when continuous variables were categorized
		(*c*) If relevant, consider translating estimates of relative risk into absolute risk for a meaningful time period
Other analyses	17	**X** Report other analyses done—eg analyses of subgroups and interactions, and sensitivity analyses
Discussion		
Key results	18	**X** Summarise key results with reference to study objectives
Limitations	19	**X** Discuss limitations of the study, taking into account sources of potential bias or imprecision. Discuss both direction and magnitude of any potential bias
Interpretation	20	**X** Give a cautious overall interpretation of results considering objectives, limitations, multiplicity of analyses, results from similar studies, and other relevant evidence
Generalisability	21	**X** Discuss the generalisability (external validity) of the study results
Other information		
Funding	22	**X** Give the source of funding and the role of the funders for the present study and, if applicable, for the original study on which the present article is based

## Abbreviations

CA: Cerebral autoregulation
ABP: Arterial blood pressure
ABP_BASELINE_: Pre-operative arterial blood pressure
LLA: Lower limit of autoregulation
NIRS: Non-invasive near-infrared spectroscopy
rSO_2_: Regional cerebral oxygen saturation
CBF: Cerebral blood flow
COx: Cererbal oximetry index
ABP_OPT_: Optimal arterial blood pressure
MRI: Magnetic resonance imaging
EtCO_2_: End-tidal carbon dioxide
NMBA: Neuromuscular blockage agents
GAM: Generalized additive method
ARIMA: Autoregressive integrated moving average
ACF: Autocorrelation function
KPSS: Kwiatkowski–Phillips–Schmidt–Shin
ADF: Augmented Dickey–Fuller
AIC: Akaike information criterion
BIC: Bayesian information criterion
LME: Linear mixed effect
TBI: Traumatic brain injury
ULA: Upper limit of autoregulation
